# Attenuation of HECT-E3 ligase expression rescued memory deficits in 3xTg-AD mice

**DOI:** 10.3389/fnagi.2022.916904

**Published:** 2022-07-29

**Authors:** Pavithra Suresh, Sureka Jasmin, Yun Yen, Hao-Jen Hsu, Peeraporn Varinthra, Tanita Pairojana, Chien-Chang Chen, Ingrid Y. Liu

**Affiliations:** ^1^Institute of Medical Sciences, Tzu Chi University, Hualien City, Taiwan; ^2^Department of Molecular Biology and Human Genetics, Tzu Chi University, Hualien City, Taiwan; ^3^Ph.D. Program for Cancer Molecular Biology and Drug Discovery, College of Medical Science and Technology, Taipei Medical University, Taipei City, Taiwan; ^4^Graduate Institute of Cancer Biology and Drug Discovery, College of Medical Science and Technology, Taipei Medical University, Taipei City, Taiwan; ^5^TMU Research Center of Cancer Translational Medicine, Taipei Medical University, Taipei City, Taiwan; ^6^Cancer Center, Taipei Municipal WanFang Hospital, Taipei City, Taiwan; ^7^Center for Cancer Translational Research, Tzu Chi University, Hualien City, Taiwan; ^8^Department of Life Sciences, Tzu Chi University, Hualien City, Taiwan; ^9^Institute of Biomedical Sciences, Academia Sinica, Taipei City, Taiwan

**Keywords:** Alzheimer's disease, 3xTg-AD, HECT-E3 ligase, learning and memory, autophagy, NLRP3 inflammasome

## Abstract

Alzheimer's disease (AD) is one of the most common progressive neurodegenerative disorders that cause deterioration of cognitive functions. Recent studies suggested that the accumulation of inflammatory molecules and impaired protein degradation mechanisms might both play a critical role in the progression of AD. Autophagy is a major protein degradation pathway that can be controlled by several HECT-E3 ligases, which then regulates the expression of inflammatory molecules. E3 ubiquitin ligases are known to be upregulated in several neurodegenerative diseases. Here, we studied the expressional change of HECT-E3 ligase using M01 on autophagy and inflammasome pathways in the context of AD pathogenesis. Our results demonstrated that the M01 treatment reversed the working memory deficits in 3xTg-AD mice when examined with the T-maze and reversal learning with the Morris water maze. Additionally, the electrophysiology recordings indicated that M01 treatment enhanced the long-term potentiation in the hippocampus of 3xTg-AD mice. Together with the improved memory performance, the expression levels of the NLRP3 inflammasome protein were decreased. On the other hand, autophagy-related molecules were increased in the hippocampus of 3xTg-AD mice. Furthermore, the protein docking analysis indicated that the binding affinity of M01 to the WWP1 and NEDD4 E3 ligases was the highest among the HECT family members. The western blot analysis also confirmed the decreased expression level of NEDD4 protein in the M01-treated 3xTg-AD mice. Overall, our results demonstrate that the modulation of HECT-E3 ligase expression level can be used as a strategy to treat early memory deficits in AD by decreasing NLRP3 inflammasome molecules and increasing the autophagy pathway.

## Introduction

Alzheimer's disease (AD) is characterized by excessive accumulation of β-amyloid protein, neurofibrillary tangles, and inflammatory molecules in various brain regions. Numerous studies have shown that neuroinflammation induced via inflammasome plays a critical role in the development of AD (Boland et al., 2008; Liu and Li, 2019; Zhang et al., 2021). The inflammasome is a protein complex consisting of three major proteins, including NLRP3 (NLR family pyrin domain containing 3), pro-caspase-1, and the adaptor protein apoptosis-associated Speck-like protein containing a CARD (caspase activation and recruitment domain) (ASC). In addition, the inflammasome regulates the release of cytokines by activating interleukin 1 beta (IL-1β). Notably, the upregulation of NLRP3 inflammasome-produced IL-1β in AD patients' brains (Kálmán et al., 1997; Benzing et al., 1999; Griffin et al., 2000) can lead to cognitive dysfunction. Thus, the NLRP3 inflammasome pathway could be a therapeutic target for AD.

Several inflammatory diseases such as Alzheimer's disease, Multiple sclerosis, and Traumatic brain injury are known to be associated with upregulation of NLRP3-inflammasome by the autophagy pathway (Moossavi et al., 2018; Holbrook et al., 2021; Zhao et al., 2021). Autophagy is a significant protein maintenance and degradation pathway that clears misfolded proteins and helps maintain homeostasis of synaptic transmission in the brain, which is tightly related to cognitive functions (Hylin et al., 2018; Hoffmann et al., 2019; Wang et al., 2019a). The autophagy process starts with the formation of the autophagosome. Beclin-1 acts as an initiator molecule for organizing the autophagosome, which is followed by vesicle elongation through lipidation of LC3 (microtubule-associated protein light chain 3B) by ATG (Autophagy related gene) complex (Chen et al., 2019). Ubiquitinated proteins and inclusion bodies are recruited into these autophagosomes by P62/SQSTM1 for degradation (Mizushima, 2007). A higher level of P62 could indicate autophagy dysfunction and protein accumulation. Beclin-1 is known to be decreased in the early stage of the AD patients' brains compared to the control group (Nixon et al., 2005; Pickford et al., 2008), suggesting a decline in autophagosome formation. In the animal model of cerebral ischemia, induction of autophagy by suppressing NLRP3 inflammasome is beneficial (Wang et al., 2019b). Besides, in the atg7 knockout mice, reduction of autophagy enhanced the activation of NLRP3 (Cho et al., 2014). Based on these studies, it is evident that maintaining homeostasis between autophagy-NLRP3 pathways could help prevent the pathogenesis of inflammatory-related diseases.

E3 ligases are classified into three subfamilies based on their substrates: RING (Really Interesting New Gene), HECT (Homologous to the E6-AP Carboxyl Terminus), and RBR (RING-Between RING-RING). Among these three families, only HECT possesses intrinsic catalytic activity. Specifically, HECT-E3 ligases can regulate autophagy by polyubiquitinating upstream molecules such as mTORC1 (mammalian target of rapamycin complex 1), ULK1 (Unc-51-like autophagy activating kinase), and beclin-1. Members of the HECT-E3 ligases family, such as NEDD4 (neuronal precursor cell-expressed developmentally downregulated 4) and WWP1 (WW domain containing E3 ubiquitin protein ligase 1), are known to directly target and degrade autophagy molecules to disrupt the autophagy process. These molecules also individually play a critical role in hippocampal-dependent learning and memory, such as fear and spatial memory (Pérez-Villegas et al., 2020). In addition, HECT-E3 ligases can regulate NLRP3 expression levels through post-translational modification. Thus, inducing the autophagy process and reducing inflammasome formation could be a new strategy to treat AD (Zhao et al., 2021).

Based on the relationship of E3 ligases with autophagy and inflammasomes in connection with the development of AD, we thus hypothesize that inhibiting E3 ligases expression can help induce the autophagy process as well as reduce the protein expression of inflammasome molecules, which in turn, enhances the memory performance in AD. A previous study has shown that the M01 compound can act as a potential HECT-E3 ligase inhibitor (Liu et al., 2017). Therefore, we decided to use M01 to modulate the HECT-E3 ligase expression with an attempt to investigate the changes in autophagy, inflammasome-related molecules, and memory performance in 3xTg-AD mice. Our results demonstrate that the administration of M01 effectively helped recover both working and reversal memory deficit in 3xTg-AD mice, accompanied by increased expression of autophagy molecules and decreased NLRP3 protein levels in the hippocampus. In addition, we detected a significant reduction in activated astrocyte levels in the M01-treated 3xTg-AD mice. Additionally, the Protein docking analysis indicates that among the HECT family members, the WWP1 and NEDD4 E3 ligases have the highest binding affinity to M01, which was later confirmed with the western blot analysis. These findings help give a better understanding of the homeostasis between autophagy and inflammation processes in the memory performance of AD. The overall result suggests that targeting modulation of E3 ligase expression could be a promising approach for treating early memory deficit in AD.

## Materials and methods

### Animals

Six-month-old 3xTg-AD (triple transgenic) and wild-type (WT) female mice were used in this study. 3xTg-AD mice were sourced from the Jackson Laboratory (Stock #34830 JAX) and maintained in the Laboratory Animal Center of Tzu Chi University for more than 10 generations. 3xTg-AD mice consist of mutations of APPSwe, PSEN1 M146, and Tau P301L. The animals were maintained under standardized conditions in a 12 h (light/dark) cycle with free access to food and water *ad libitum*. We executed all animal experiments according to the protocol approved by the Institutional Animal Care and Use Committee (IACUC). The approval number is #109047.

### M01 preparation and treatment

M01 was synthesized as previously described (Liu et al., 2017). M01 was dissolved in saline: DMSO at a ratio of 9:1. The compound was orally delivered using gavage (ST-F172 0.9 mm x L 50 mm, 20G, Shineteh Instruments Co., Ltd.) 2 h before behavior tests.

### Open field test (OFT)

To evaluate animals' exploratory behavior and locomotor activity, we performed an open field test (OFT) using the previously published protocol (Phasuk et al., 2021b). The open field chamber consists of a white base surrounded by four black walls measuring 50 cm (L) × 50 cm (W) × 50 cm (H). The speed and distance traveled in the chamber were recorded using a video camera and scored by automated software (EthoVision XT 15, Noldus Information Technology) for a 10 min session.

### T-Maze—spontaneous alteration

Spontaneous alteration is a natural tendency of mice to alternate free choices in the T-maze through trials. To assess mice's spatial/working memory and alteration behavior, we performed T-maze-spontaneous alteration indicating mice's ability to differentiate between the novel and familiar arms. Mice with healthy cognition tend to spend more time in the novel arm (Rosenzweig et al., 2019). The T-maze consists of a white non-transparent board with three arms—a long tail measuring 30 cm and right and left arms (each 30 cm) connected to the tail at perfect right angles. In the trial session for 5 min, the animal was allowed to access only one arm (right familiar) by blocking the other (left novel) with a sliding door. In the test session (5 min) carried out after the trial session, the block was removed from the left arm, and the animal was allowed to access both arms freely. The time spent by the mice in both arms, latency to the novel arm, and alteration percentage to the novel arm [frequency to novel arm/(total frequency) multiplied by 100] were recorded and measured by automated software (EthoVision XT 15, Noldus Information Technology).

### Morris water maze (MWM)

The Morris water maze procedure was adapted from the published protocol (Phasuk et al., 2021a). The Morris water maze test used a white, non-transparent tank 39 cm in height and 100 cm in diameter. The water-filled tank was maintained at 21 ± 1°C and made opaque by mixing non-toxic white paint (Cat. # 187203, Palmer paint products, USA). The pool was divided into four equal quadrants with a different cues. During visible training (day 1 and 2), a circular platform 5 cm in diameter was placed 1 cm above the water in a different quadrant in each trial, and the mice were placed on the platform for 20 s to allocate the cues. Then, the mice were allowed to explore the pool for 60 s. Mice were removed when they reached the platform or could not find the platform within 60 s. There were a total of five trials with inter-trial intervals of 30 min. During the hidden platform test, the platform was kept 1 cm below the water in the target quadrant, and the entrance point of mice was changed in each trial to avoid track memorization. The mice were given five trials a day to find the hidden platform with a trial period of 60 s for four consecutive days. The probe test was carried out on the 7th day, on which the platform was removed from the pool. The mice were placed through unfamiliar entrance points and allowed to swim freely for 60 s. On the following day of the probe test, a reversal-learning session was conducted with the platform opposite to the acclimatized target quadrant and five trials were performed (Arqué et al., 2008; Karabeg et al., 2013). A video tracking system (EthoVision XT 15, Noldus Information Technology) was used to measure the path length, swimming speed, escape latency, and percentage of time spent in each quadrant.

### Immunoblotting analysis

The hippocampus was dissected from the whole brain for immunoblotting and lysed using 1X RIPA buffer (Millipore, USA) supplemented with protease and phosphatase inhibitors. The lysate was cleared by centrifugation at 13,500 rpm for 15 min at 4°C, and the supernatant was stored at −80°C for further experiments. The protein concentration was estimated by Bradford's method every time before gel electrophoresis. The protein samples were prepared in reducing agent (NuPAGE^®^ Invitrogen) + LDS sample buffer (4X – NuPAGE^®^ Invitrogen) and boiled at 95°C for 10 min. A 50 μg protein sample was separated by SDS–PAGE in an 8 or 12% polyacrylamide gel and transferred to a PVDF membrane (0.45 μm pore size) at 25 volts in a cold room overnight. The membrane was then blocked with 5% milk/1% BSA in tris buffered saline with 0.1% Tween 20 (TBST) for 1 h at room temperature. After blocking, primary antibodies against NLRP3 (1:1,000; Abcam, UK), Beclin-1 (1:1,000; Abcam, UK), P62 (1:1,000; Abcam, UK), Caspase 1 (1:1,000; Cell Signaling Technology, USA), LC3B (1:1,000; Abcam, UK), WWP1 (1:1000; Abnova), NEDD4 (1:1000; Merck millipore), and anti-β-actin (1:10,000; Sigma–Aldrich, USA) diluted in TBST with 0.1% BSA were added and incubated overnight in a shaker. The blots were transferred to appropriate secondary antibody conjugates diluted in 0.1% milk for 1 h, developed using ECL (Western lighting^®^ Plus ECL, PerkinElmer Inc, MA, USA) and detected under a Bio-Rad ChemiDoc MP High-performance Cold Light Fluorescence Analysis system or High sensitivity biomedical imaging system chemstudio plus. The quantification of band intensities was performed using ImageJ version 1.52a (National Institutes of Health, USA).

### Immunofluorescence staining and image analysis

The brains were collected from mice through cardiac perfusion using 4% paraformaldehyde (PFA) and 0.9% saline in PBS, kept in PFA for 48 h and then transferred to 30% sucrose. The brains were sliced into thin sections with 35 μm thickness using cryostat. The sections were permeabilized in permeating buffer (1% Triton + 2% Tween in PBS) for 30 min and blocked with 1% normal goat serum (NGS) + 0.3% Triton X-100 for 1 h at room temperature (RT). After blocking, primary antibodies against NLRP3 (1:100; Abcam, UK), Beclin-1 (1:100; Abcam, UK), P62 (1:100; Abcam, UK), and GFAP (1:200; Abcam, UK) were added and incubated overnight at 4°C. Respective secondary antibodies were added and incubated for 1 h at RT after washing the sections with 0.25% Triton+ PBS three times for 5 min each. DAPI (4'-6-Diamidino-2-phenylindole) was used to stain the DNA. The sections were mounted on 76 × 26 mm adhesive glass slides and covered with coverslips for imaging. Upright Conjugate Focus Microscope NIKON C2si+ confocal microscope was used to capture images with 10×, 20×, and 40× objective lenses under 405,488, and 561 nm wavelengths. All images were adjusted and cropped using ImageJ version 1.52a (National Institutes of Health, USA). The calculation was done using ImageJ software, 3–5 fields (200 × 200 μm) from 3 to 4 sections per mouse were used to calculate the positive area percentage of each antibody.

### Electrophysiology

Extracellular recording of the CA1-CA3 Schaffer collateral synapse was performed as previously reported (Varinthra et al., 2021). The brains were removed with decapitation and immediately placed in ice-cold artificial cerebrospinal fluid (ACSF). Coronal sections of the hippocampus 350 μm thick were obtained in oxygenated ACSF using a vibrating microtome (Microslicer DTK-1000, Dosaka EM Co. Ltd., Kyoto, Japan). Slices were maintained in oxygenated ACSF with 95% O_2_/5% CO_2_ at 28–30°C for at least 2 h before transferring to the recording chamber. In the recording chamber, the brain slice was supplied with oxygen (O_2_)-concentrated ACSF at a speed of 2–3 mL/min at 28°C. Field excitatory postsynaptic potentials (fEPSPs) were recorded from the CA1 stratum radiatum region of the hippocampus via glass micropipettes (PC-10 Needle Puller, Narishige, Japan) filled with ACSF. Stimulation was provided by stainless steel unipolar electrodes (Frederick Haer Company, Bowdoinham, ME, USA). Baseline stimulus intensity was set at the level that evoked 30–40% of the maximum fEPSPs response as determined from the input-output curve. Baseline fEPSPs were recorded for 20 min followed by high-frequency stimulation (HFS) with 3 trials of 100 pulses at 100 Hz for 60 s. The fEPSPs were stimulated every 20 s and recorded for 80 min. The signals were amplified by an Axon Multiclamp 700B amplifier, filtered at 1 kHz and digitized at 10 kHz by a CED MicroPower 1401 MKII interface (Cambridge Electronic Design, Cambridge, UK) using Signal software. The slope of fEPSPs is measured using Axon pCLAMP 11 electrophysiology data acquisition and analysis software.

### Protein docking analysis

To predict the interaction between selected HECT-E3 ligases and M01, the DOCK module with “Induced fit” refinement in the MOE2020.09 software program (https://www.chemcomp.com) was used for molecular docking. M01 was manually built in the MOE software package to dock with different E3 ligases (PDB: 5C91, 3TUG, 1ND7, 3H1D, 5HPK with M01) for comparison. MOE software was used to eliminate the water molecules, and missing short loops were added. The binding free energy of the ligand to the receptor was estimated using GBVI/WSA ΔG, a force field-based scoring function. The scoring function was used to predict the binding affinity between M01 and selected E3 ligases. The lowest binding score represents better affinity.

### Statistical analysis

All statistical analyses were performed using IBM SPSS 22 statistical software, and all values are presented as the mean ± SEM. One-way ANOVA was conducted to compare between groups. In addition, path length, speed, and latency during hidden platform trials and time spent during the probe test were analyzed using mixed-design repeated measure ANOVA with trials and groups as within-subjects and between-subjects, respectively. For electrophysiological recording, one-way ANOVA followed by Tukey's *post hoc* test was applied for multiple comparisons. A *p* < 0.05 was considered statistically significant. ANOVA tables for western and IHC are shown in Supplementary Table 1. All graphs were plotted with GraphPad Prism 8.0 software.

## Results

### E3 ligase inhibitor, M01, rescued working memory deficits in 3xTg-AD mice

**Figure 1:**
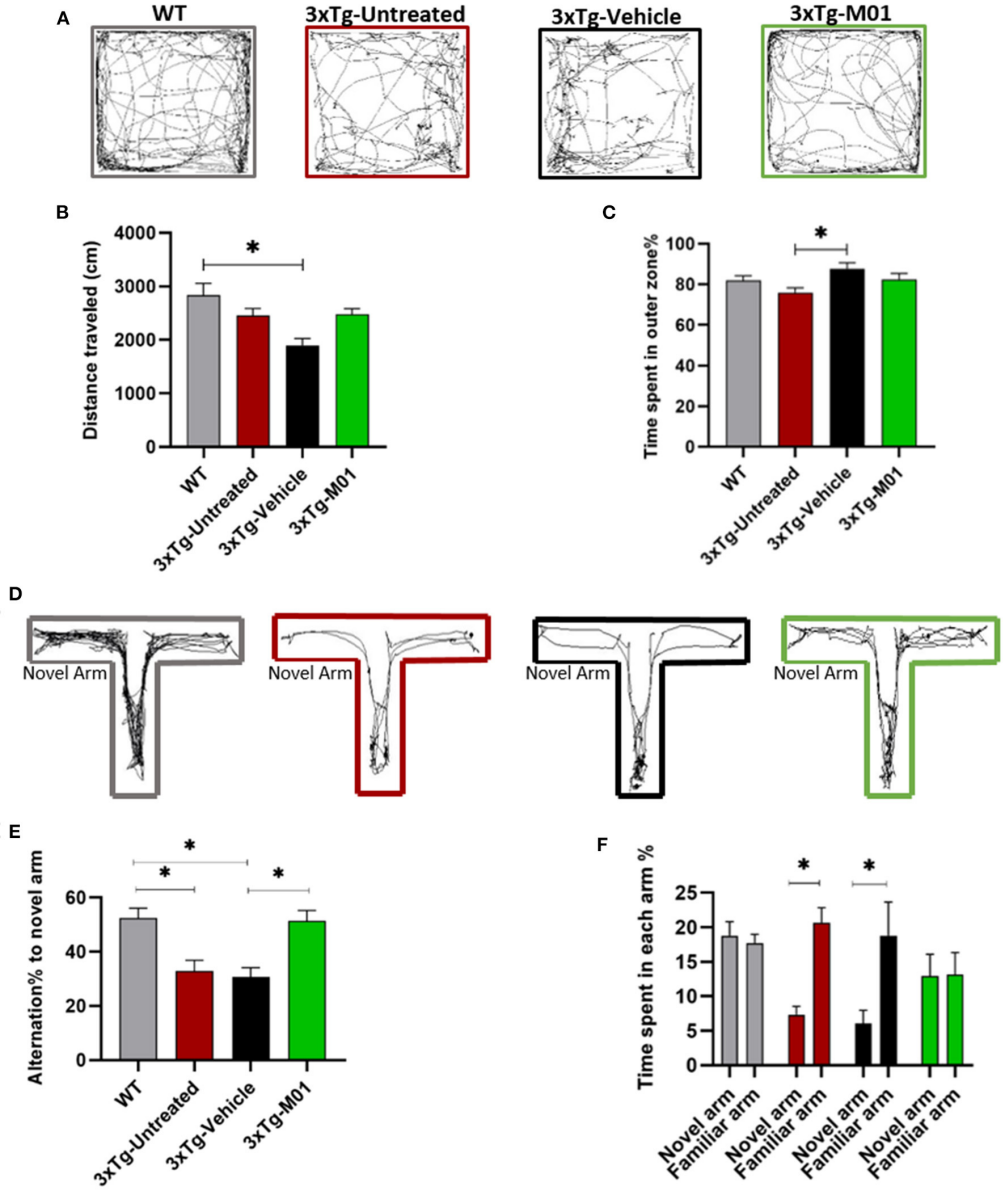
M01 rescued working memory deficits in 3xTg-AD mice. **(A)** Trace diagram of mice in the OFT. **(B)** distance traveled in the OFT shows difference between WT and 3xTg-vehicle group, and no statistical difference between 3xTg-M01 group. **(C)** Time spent in the outer zone by each group of mice shows significant difference between 3xTg- untreated and vehicle group but not with the other groups **(D)** Trace pattern of all the groups in the T-maze paradigm. **(E)** Quantification data represent the percentage of alternation frequency toward the novel arm. Statistical difference was observed between WT and 3xTg- untreated, vehicle groups. M01 treated 3xTg mice shows high alternation toward novel arm compared to 3xTg-Vehicle group. **(F)** Percentage of time spent in the novel and familiar arms. 3xTg-untreated and vehicle groups spent significantly more time in the familiar arm while WT and M01 groups explored both the arms equally. Data are expressed as the mean ± SEM. *p < 0.05, *n* = 9–15/group, and one-way ANOVA followed by Tukey's test were used to compare between groups.

Carroll et al. (2010) found that female 3xTg mice exhibit higher Amyloid-beta and behavior deficits than age-matched controls (Carroll et al., 2010). (Stimmell and Baglietto-Vargas, 2019) found that 6-month-old female 3xTg mice showed spatial orientation deficit and higher pathology, specifically in the hippocampus, compared to male mice (Stimmell and Baglietto-Vargas, 2019). Therefore, we decided to use female 3xTg mice to study AD pathogenesis in our study. DMSO has been demonstrated to have an effect on animal behavior (Penazzi et al., 2017). However, the M01 dissolves better in DMSO; and therefore, we design a DMSO only group (the vehicle group) to evaluate the possible effect of DMSO on our study. We first performed an OFT to evaluate the effect of M01 on locomotor activity. Moving trace patterns were shown in the OFT paradigm (Figure 1A). No significant difference was found in moving distance (Figure 1B) between WT, 3xTg-untreated, and 3xTg-M01 but the 3xTg-vehicle group traveled less distance than other groups [*F*_(3, 30)_ = 6.07, *p* < 0.05]. Time spent in the outer zone shows a difference between 3xTg-untreated and vehicle groups [*F*_(3, 30)_ = 3.765, *p* < 0.05, Figure 1C) but shows no difference when compared with the WT and 3xTg-M01 groups.

To further determine the effect of M01 on working memory, we performed a T-maze test on all four groups. Normal mice usually spend a longer period of time exploring the novel arm or equal time in the novel and familiar arms. Here, 3xTg-untreated and vehicle groups show less alternation frequency toward the novel arm. However, the frequency of visiting the novel arm increased significantly in the M01-treated 3xTg group (*F*_(3, 50)_ = 9.940, *p* = 0.000, Figure 1E), which is also evident from the tracing pattern (Figure 1D). We also found that both the 3xTg-untreated [*t*_(28)_ = 3.651, *p* = 0.001] and vehicle groups [*t*_(22)_ = −2.433, *p* = 0.024] spent a longer time in the familiar arm than the novel arm (Figure 1E). After the M01 treatment, 3xTg mice explored the familiar and novel arms equally [*t*_(20)_ = −0.43, *p* = 0.966] similar to WT [*t*_(30)_ = 0.445, *p* = 0.659] (Figure 1F). The 3xTg untreated and vehicle group spending more time in the familiar arm could be because of the anxiety and also working memory deficit. The 3xTg mice have been shown to exhibit anxiety-like behavior (Giménez-Llort et al., 2013; Pietropaolo et al., 2014).

### E3 ligase inhibitor, M01, treatment rescued spatial reversal learning deficit in 3xTg-AD mice

**Figure 2:**
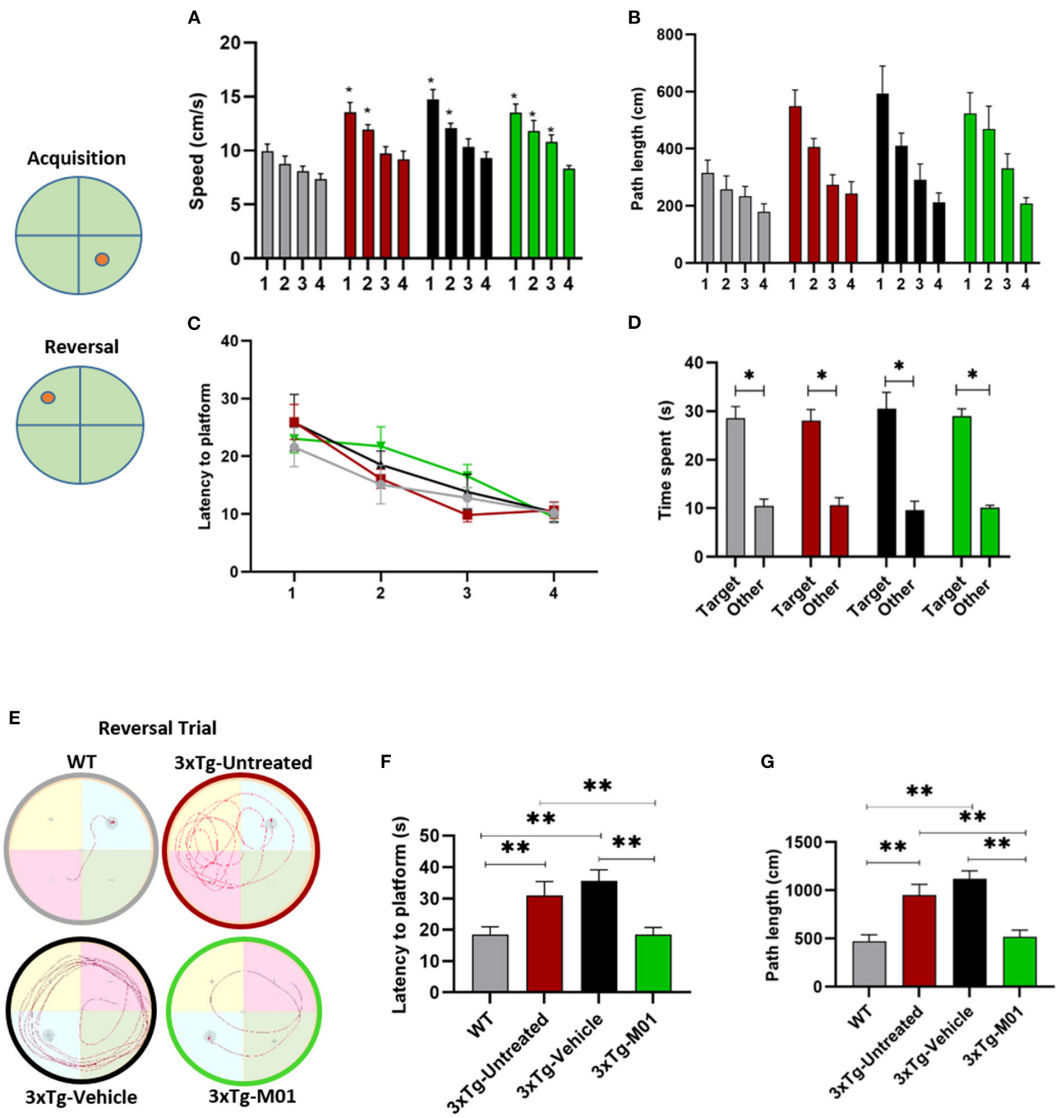
M01 rescued working memory and reversal learning deficits in 3xTg-AD mice. Schematic representation of the platforms during acquisition and reversal trials. **(A)** swimming speed during the hidden platform training days, a significant decrease in the swimming speed was recorded throughout the training days in 3xTg- untreated, -vehicle, and M01 groups. **(B)** path length, and **(C)** latency to reach the platform during hidden platform days also decreased over the training days. **(D)** Time spent in the platform quadrant during the probe test by each group. All groups spent a significantly longer time in the target quadrant. A reversal trial was used to measure learning flexibility. **(E)** Trace diagram during the reversal trial **(F)** latency to reach the platform, and **(G)** path length during the reversal. WT and M01 treated 3xTg mice spent significantly less time finding the platform. The results are plotted as mean ± SEM. *p < 0.05, **p < 0.01, *n* = 8–12/group. Repeated measures ANOVA followed by Tukey's *post hoc* test were used to compare between groups.

Next, we investigated spatial memory using the Morris water maze (MWM). Throughout the training course, all the groups performed similarly. In the acquisition phase, by using mixed-design repeated ANOVA, we found that there was a significant difference observed between hidden platform days for swimming speed [*F*_(3, 99)_ = 35.66, *p* = 0.000, Figure 2A], path length [*F*_(3, 99)_ = 33.35, *p* = 0.000, Figure 2B] and latency to find the platform [*F*_(3, 99)_ = 17.31, *p* = 0.000, Figure 2C], while no interaction was observed between days and groups. These results indicated that all four groups can learn the task during the acquisition trials. During the reference memory test, all the groups spent longer time in the target quadrant than in other quadrants (Figure 2D). These data indicated that the spatial memory is still intact in 6-month-old 3xTg-AD mice.

During the reversal trial day, 3xTg-untreated and vehicle groups took longer to find the platform, while the WT and 3xTg-M01 groups performed significantly better. Throughout the reversal learning trials, the 3xTg-M01 group spent less time finding the newly positioned platform (latency) **[***F*_(3, 33)_ = 6.69, *p* < 0.05, Figure 2F], and the path length for them to reach the platform was also decreased [*F*_(3, 33)_ = 11.90, *p* < 0.05, Figure 2G] compared with that of the 3xTg-Untreated and vehicle groups. In addition, the swimming trace patterns of the WT and the M01 treated groups were significantly different during MWM reversal trials (Figure 2E). All behavior experiments were performed 2 h after M01 treatment. For Morris water maze, the M01 was administered 2 h before the experiment every day. Therefore, the recorded behavioral impairment of the reversal trial should be the cumulative effect of the 8-day M01 treatment. These results demonstrated that M01 treatment effectively rescues early symptoms of working memory and reversal learning deficits in the 3xTg-AD mice.

### E3 ligase inhibitor, M01, administration leads to increased protein levels of autophagy molecules and decreased NLRP3 inflammasome molecules in 3xTg-AD mice

**Figure 3:**
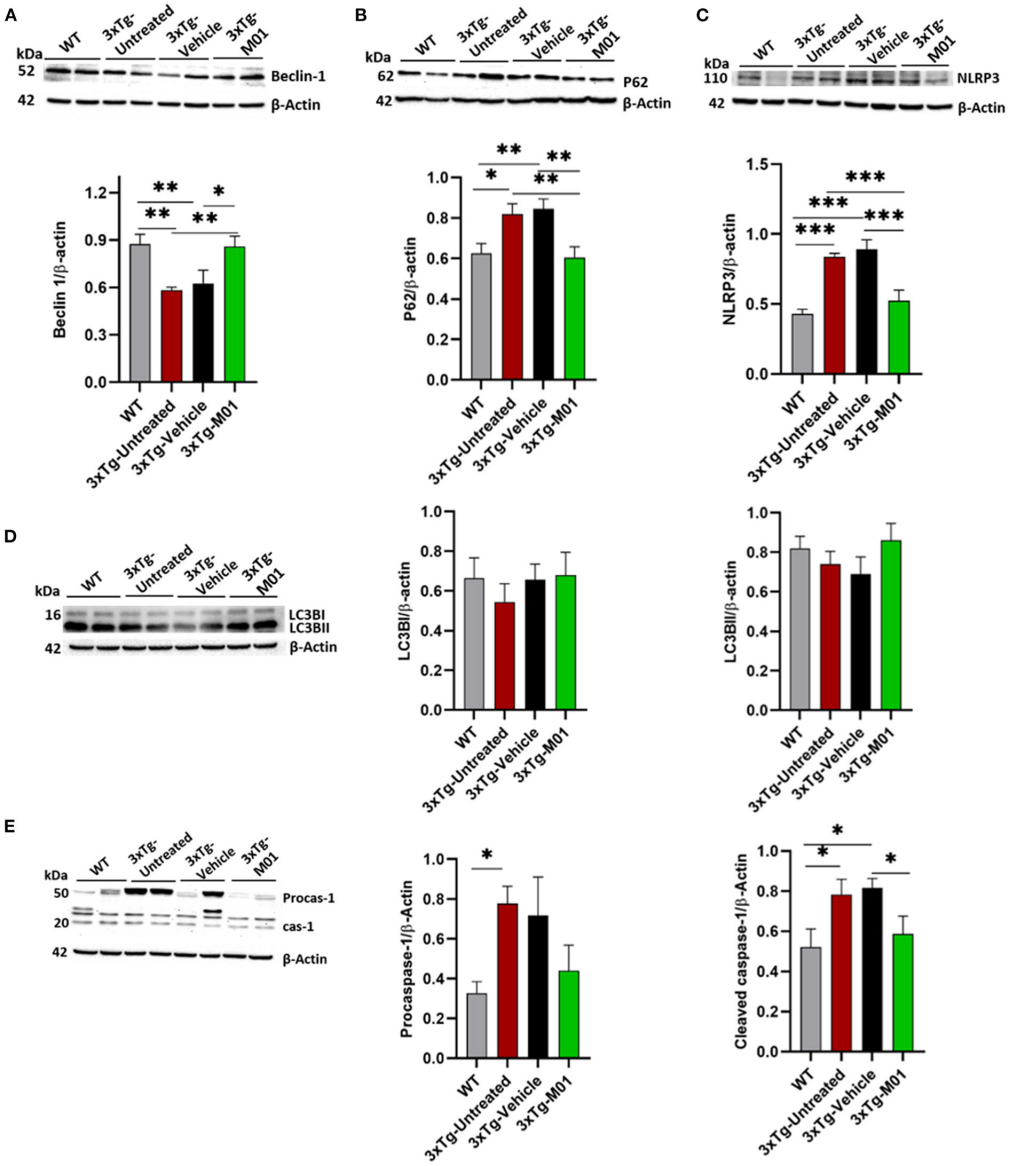
M01 administration increased the protein expression levels of autophagy-related molecules and decreased NLRP3 in the hippocampus. Immunoblots and quantification graphs of proteins **(A)** beclin-1expressed significantly less in 3xTg-untreated and vehicle groups compared to WT and M01 groups. **(B)** P62 expression is high in 3xTg-untreated and vehicle groups, and M01 decreased the P62 to WT level. **(C)** NLRP3 over-expressed in 3xTg-untreated and vehicle group, M01 administration significantly decreased NLRP3 level. **(D)** LC3BI&II shows no statistical difference between groups. **(E)** Pro-caspase-1 expressed significantly higher in 3xTg-untreated group compared to WT group. Cleaved caspase-1 expressed significantly higher in the 3xTg-untreated and -vehicle groups; M01 treatment significantly decreased the caspase-1 level in the hippocampus. The results are plotted as the mean ± SEM. *p ≤ 0.05, **p < 0.01, ***p < 0.001, *n* = 6–8/group for western blot analysis.

**Figure 4:**
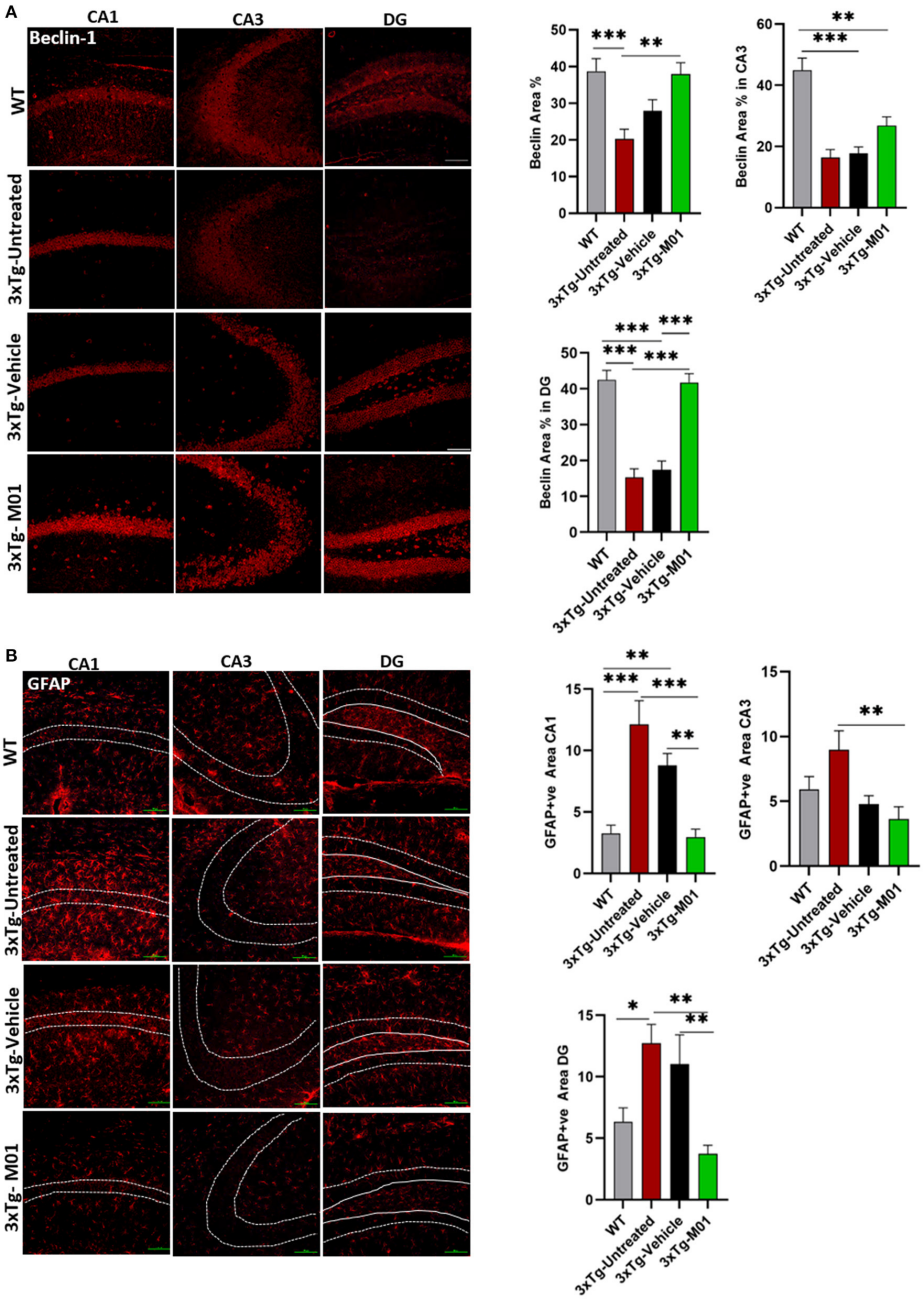
M01 administration increased beclin-1 and decreased GFAP expression levels in the hippocampus of 3xTg-AD mice. **(A)** The expression of Beclin-1 in CA1, DG, and CA3 significantly decreased in the 3xTg-untreated and -vehicle groups. M01 treatment increased the expression in CA1 and DG. The quantification graphs are demonstrated on the right panels. **(B)** The expression of GFAP in CA1, CA3, and DG of the hippocampus was significantly increased in 3xTg-untreated and -vehicle groups. M01 treatment significantly decreased the GFAP expression. The quantification graphs are demonstrated on the right side. Scale bar: 100 μm. The results are plotted as the mean ± SEM. *p < 0.05, **p < 0.01, ***p < 0.001 and *n* = 3–4 mice/group and 3 sections/mice for IF.

We further investigated the changes in the protein levels of autophagy and inflammasome-related molecules after the M01 administration to 3xTg mice. As shown in Figure 3A, autophagy initiator molecule beclin-1 [*F*_(3, 26)_ = 5.763, *p* < 0.05] was downregulated in both the 3xTg-untreated (*p* < 0.01) and vehicle group (*p* < 0.01). After the administration of M01, we detected that beclin-1 protein levels were significantly increased in the hippocampus of the 3xTg-M01 group. We further confirmed the result using the immunofluorescencent staining technique on the hippocampus and found that beclin-1 was upregulated in the M01 treated group in CA1 [*F*_(3, 57)_ = 8.993, *p* = 0.000], CA3 [*F*_(3, 76)_ = 19.379, *p* = 0.000] and DG [*F*_(3, 83)_ = 33.390, *p* = 0.000] when compared to the 3xTg-untreated and vehicle groups (Figure 4A). We also investigated the LC3 protein involved in the elongation and formation of autophagosomes, and found that the expression levels of LC3BI [*F*_(3, 24)_ = 0.354, *p* = 0.787] and LC3BII protein [*F*_(3, 24)_ = 1.036, *p* = 0.392] were not significantly different between the groups (Figure 3D). We then checked the autophagy substrate and reporter molecule P62 and noted that it was upregulated in the 3xTg-untreated and vehicle groups indicating that the attenuation of autophagy has occurred [*F*_(3, 24)_ = 6.361, *p* < 0.01, Figure 3B]. On the other hand, the M01 treatment reduced the P62 protein expression (*p* = 0.785) to the WT level which indicated that the autophagy functions at the normal level (Figure 3B).

**Figure 5:**
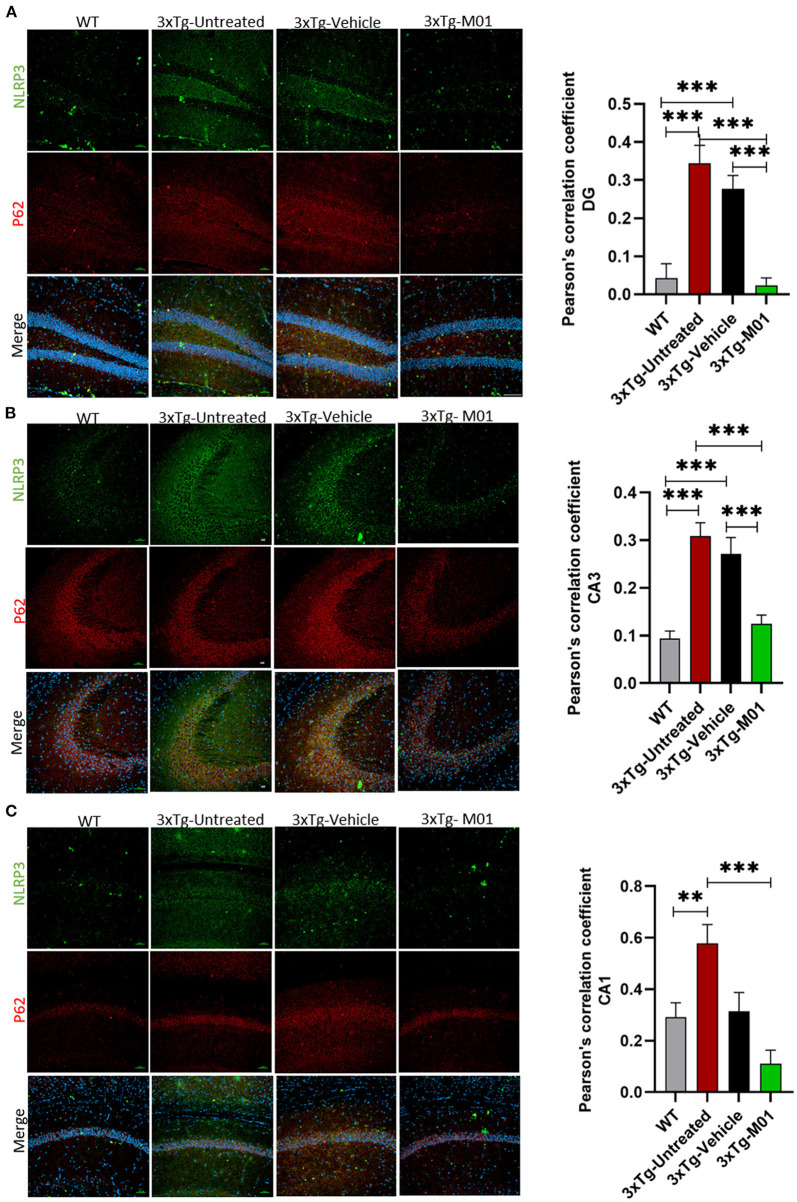
M01 administration decreased P62 and NLRP3 co-localization in the hippocampus of 3xTg-AD mice. The immunofluorescence staining NLRP3 (green), P62 (red), and merged (yellow) in **(A)** DG, **(B)** CA3, and **(C)** CA1. Co-localization of NLRP3 and P62 is significantly high in 3xTg-untreated and vehicle groups, M01 treatment decreased the co-localization of NLRP3 and p62 in the hippocampus of 3xTg mice. Pearson's correlation coefficient was applied to quantify the co-localization and graphs were plotted on the right panel. Scale bar: 100 μm. The results are presented as the mean ± SEM. **p < 0.01, ***p < 0.001 and *n* = 3–4/group and 3 sections/mice for IF.

The expression levels of NLRP3 [*F*_(3, 28)_ = 13.892, *p* < 0.000] and cleaved caspase-1 proteins [*F*_(3, 18)_ = 3.698, *p* < 0.05, Figure 3E], both of which are components of the inflammasome complex, was significantly upregulated in 3xTg-untreated and vehicle groups. M01 treatment decreased the NLRP3 inflammasome protein in the 3xTg mice (Figure 3C). We further performed immunofluorescent staining to confirm the differential expression levels and cell types of these molecules in the hippocampus and found that after treatment, 3xTg-M01 mice showed significant decrease of NLRP3 and P62 colocalization in the DG [*F*_(3, 59)_ = 23.485, *p* = 0.000, Figure 5A], CA3 [*F*_(3, 81)_ = 19.515, *p* = 0.000, Figure 5B], and CA1 [*F*_(3, 23)_ = 8.545, *p* = 0.001, Figure 5C] of the hippocampus. Thus, our results indicate that the administration of M01 can increase the surge of autophagic flux, which might have caused the decreased level of NLRP3 protein in the hippocampus of 3xTg-AD mice.

The over-activation of astrocytes can lead to the inhibition of proteasome pathways, which in turn causes the AD pathology development (Ozcelik et al., 2013), induction of autophagy reduced the astrogliosis in P301S Tau Transgenic mice. To determine whether the M01 treatment can decrease reactive astrocytes, we performed immunofluorescent staining in the hippocampus of 3xTg-AD mice. Interestingly, 3xTg-M01 group showed reduced expression of astrocytes marker, glial fibrillary acidic protein (GFAP), compared to the 3xTg-untreated and vehicle groups in CA1 [*F*_(3, 37)_ = 12.827 *p* = 0.000], CA3 [*F*_(3, 34)_ = 4.037 *p* = 0.015], and DG [*F*_(3, 33)_ = 5.103, *p* = 0.005] (Figure 4B).

### M01-treated 3xTg-AD mice exhibited enhanced long-term potentiation in the hippocampal CA1 region

**Figure 6:**
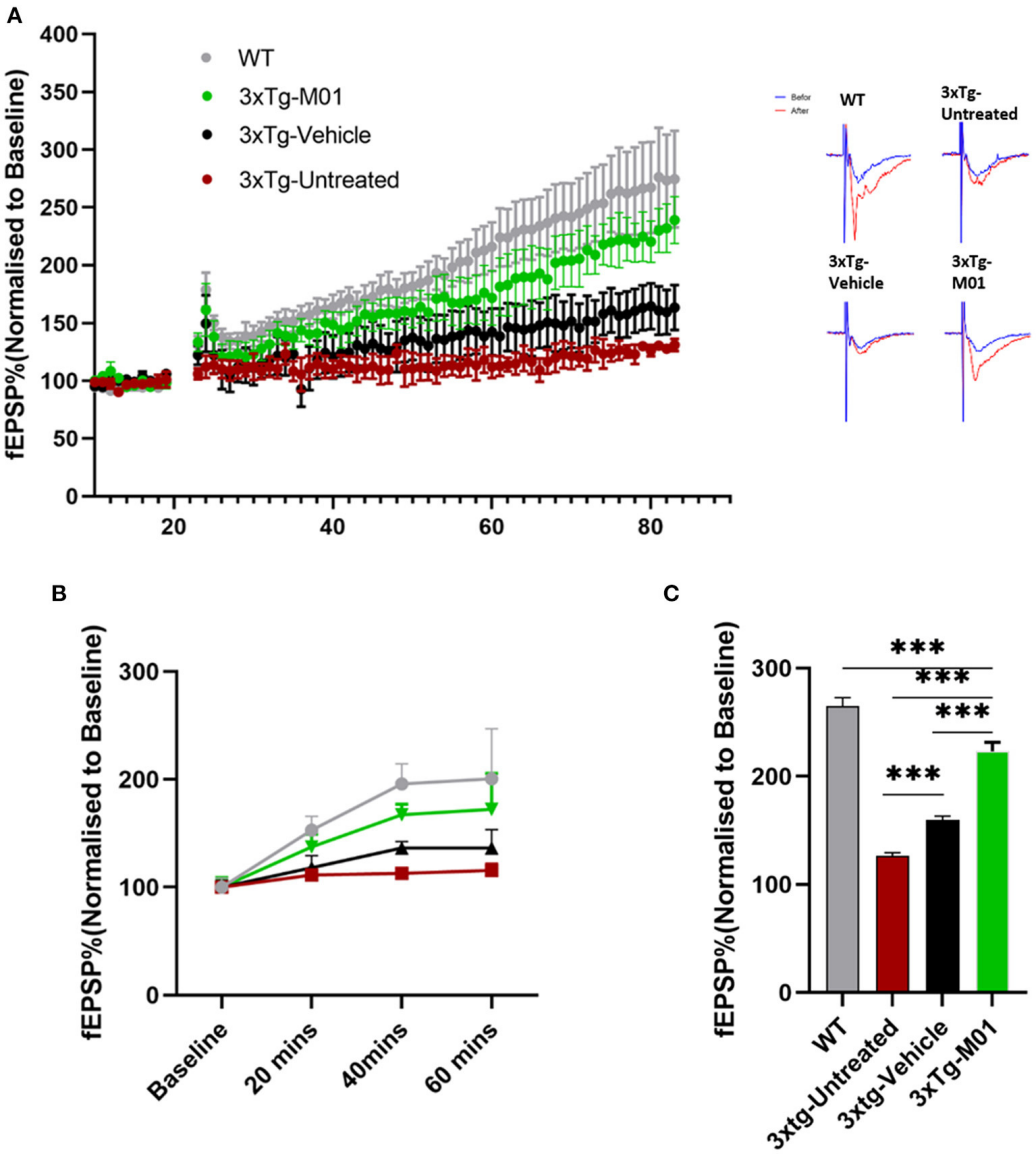
Enhanced CA1 long-term potentiation was recorded in M01-administered 3xTg-AD mice. **(A)** Average values of fEPSPs (normalized to baseline) were plotted for each group along with representative traces of LTP. **(B)** fEPSP slope plotted by different time points. **(C)** Quantitative graphs show a significant difference in LTP between WT, 3xTg-untreated, 3xTg-vehicle, and 3xTg-M01. ***p ≤ 0.000. *n* = 3–4/group. The results are plotted as the mean ± SEM, and one-way ANOVA followed by Tukey's *post hoc* test was applied for multiple comparisons.

The dysregulation of inflammatory mediators has been known to disrupt neural plasticity (Di Filippo et al., 2008). Also, we found that M01 can decrease the inflammasome in 3xTg mice. Therefore, we performed extracellular recording in CA3-CA1 Schaffer collateral synapses in the hippocampus to see the effect of M01 on neural plasticity. No difference was observed in the baseline fEPSP recorded among the groups (Figures 6A,B). We observed that early long-term potentiation (E-LTPs) of 3xTg-untreated and 3xTg-vehicle mice recorded in the Schaffer collateral stimulation were impaired [*F*_(3, 36)_ = 1438.64, *p* = 0.00, Figure 6C] when compared to the WT and M01 groups. After the administration of M01, 3xTg-M01 mice showed improved induction of LTP, and the LTP lasted throughout the recording after HFS when compared to 3xTg-untreated and 3xTg-vehicle groups (Figures 6A–C). Interestingly, we observed that the vehicle (DMSO) group shows increased fEPSP. Based on a previous study, DMSO can influence the spine density in the APP_SDL_ AD mouse model (Penazzi et al., 2017). However, 3xTg-M01 group evoked higher fEPSP than the 3xTg-untreated and vehicle groups.

### M01 has a high binding affinity with WWP1 and NEDD4 E3 ligase

**Figure 7:**
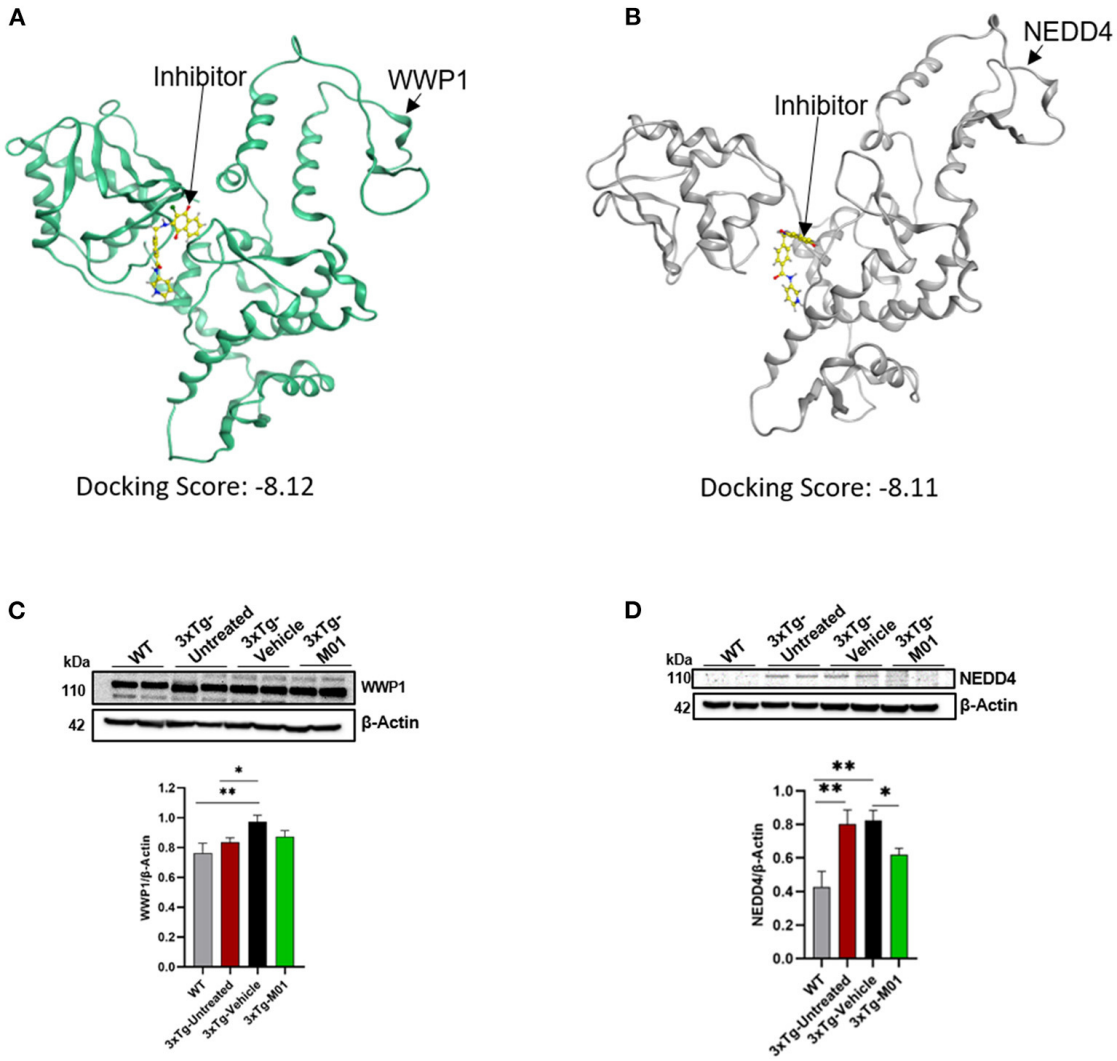
M01 has a high binding affinity with WWP1 and NEDD4 E3 ligase. Protein structure of **(A)** WWP1 (green) (PDB code: 1ND7), **(B)** NEDD4 (gray) (PDS code: 5C91) docked with M01 (yellow), **(C)** Images of western blotting of anti-WWP1, and **(D)** anti-NEDD4 antibodies against protein extraction from the hippocampus. M01 treatment significantly decreased the protein level of NEDD4 E3 ligase in 3xTg mice. The results are plotted as mean ± SEM. *p < 0.05, **p < 0.01. *n* = 5–6/ group for western blot analysis.

To find the potential target of M01, we selected five E3 ligases involved in autophagy and AD for protein docking. A docking score was given to each E3 ligase (Supplementary Figure 1), which represents the binding affinity of M01 with the target. WWP1 (Figure 7A) and NEDD4 (Figure 7B) attained the lowest docking score among the other E3 ligases, indicating that both of these E3 ligases have preferable docking pose. We performed western blotting to confirm the docking results by checking the expression levels of WWP1 (Figure 7C), and NEDD4 protein (Figure 7D) in the hippocampus. We found a significant increase in the expression level of WWP1 protein [*F*_(3, 36)_ = 3.487, *p* = 0.027] in 3xTg-vehicle group compared to the WT group. The NEDD4 [*F*_(3, 35)_ = 6.755, *p* = 0.001) protein expression level was significantly higher in 3xTg-untreated and -vehicle groups compared to the WT group. After the M01 treatment, NEDD4 (*p* < 0.05) protein expression level was significantly decreased in 3xTg-M01 group compared to the 3xTg-vehicle group.

## Discussion

The results of our study suggest that modulating HECT-E3 ligase expression level can effectively prevent memory decline in the early stage of AD, which seems to be associated with the enhancement of the autophagy process and reduced expression level of the NLRP3 inflammasome proteins. The AD drug development has recently been shifted to chronic inflammation reduction because limited success was achieved in clinical trials focusing on Aβ amyloid clearance (Morrison, 2016; Businaro et al., 2018). The accumulation of Aβ can release inflammatory cytokines (Wang et al., 2015), and impair synaptic transmission and memory performance (Karisetty et al., 2020). Here, we demonstrate that reducing the excessive inflammatory response by modulating HECT-E3 ligase to enhance the autophagy process is a promising therapeutic approach to preventing early memory decline in AD.

Autophagic dysregulation plays a critical role in the development of neurodegenerative diseases, especially AD. Studies have found that beclin-1 is downregulated in the cortex of AD patients (Pickford et al., 2008; Bieri et al., 2018). It is also reported that beclin-1 is cleaved during AD, which correlates with neuronal loss (Bieri et al., 2018), which supports the assumption that rescuing beclin-1 level to normal can decrease AD progression. In addition, a reduction in the autophagy process leads to the accumulation of inflammatory cytokines in the brain (Saitoh and Akira, 2016), while reversing autophagic function in the TgCRND8 AD mouse model improves learning and memory deficits (Yang et al., 2010). The autophagic adaptor protein P62 aids the degradation of the NLRP3 inflammasome through autophagosomes in human primary monocytes (Shi et al., 2012). Ising et al. found that NLRP3 inflammasome was activated in the cortex of frontotemporal dementia patients and Tau22 mice, a tau pathology mouse model (Ising et al., 2019). The autophagy pathway limits the NLRP3 inflammasome by eliminating inflammasome components and activating factors (Biasizzo and Kopitar-Jerala, 2020). These studies support our findings that induction of the autophagy pathway can efficiently eliminate the NLRP3 burden in AD. Glatigny et al. found that behavioral training can increase autophagy molecules in the brain (Glatigny et al., 2019) and the beclin-1 level was upregulated after the MWM and fear conditioning. Inhibiting beclin-1 in hippocampal neurons can disrupt memory performance (Glatigny et al., 2019). Also, Atsushi Sato et al. (2012) showed rapamycin, an autophagy inducer treated for 2 days could improve the social interaction and rearing in tuberous sclerosis mice. These reports demonstrate that autophagy induction is crucial for memory performance and support our approach by modulating E3 ligase expression to restore autophagy function and improve the memory performance of 3xTg-AD mice.

In our study, we used protein docking to identify the target of M01. We found that WWP1 and NEDD4 may be the primary candidates and could be responsible for the therapeutic effect of M01 on 3xTg-AD mice. NEDD4 (Kwak et al., 2012) was increased in AD pathology. Furthermore, it has been reported that NEDD4-1 (Pei et al., 2017), NEDD4-2 (Wang et al., 2016), ITCH (Chhangani et al., 2014), and WWP1 (Sanarico et al., 2018) can regulate autophagy, either negatively or positively. These reports support our findings that modulation of E3 ligase function can induce the autophagy process. Further site-direct mutagenesis or inhibitory experiments are necessary to verify the roles of WWP1 and NEDD4 in the memory performance of 3xTg-AD mice.

Synaptic strength represents the degree of memory performance. Several studies have reported the correlation between an E3 ligase and synaptic strength. Yuen et al. (2012) showed that knockdown of the E3 ligase NEDD4-1 in the prefrontal cortex rescued the loss of glutamatergic responses and recognition memory in stressed mice. Furthermore, NEDD4-1 controls the downscaling of synaptic strength by decreasing AMPARs (Scudder et al., 2014), while knockdown of NEDD4-1 enhances AMPAR-EPSCs. These studies demonstrate that targeting the E3 ligase can alter synaptic transmission in AD. Our electrophysiological recording confirms that modulation of E3 ligases can enhance synaptic plasticity via induction of excitatory postsynaptic potential in 3xTg-AD mice.

**Figure 8:**
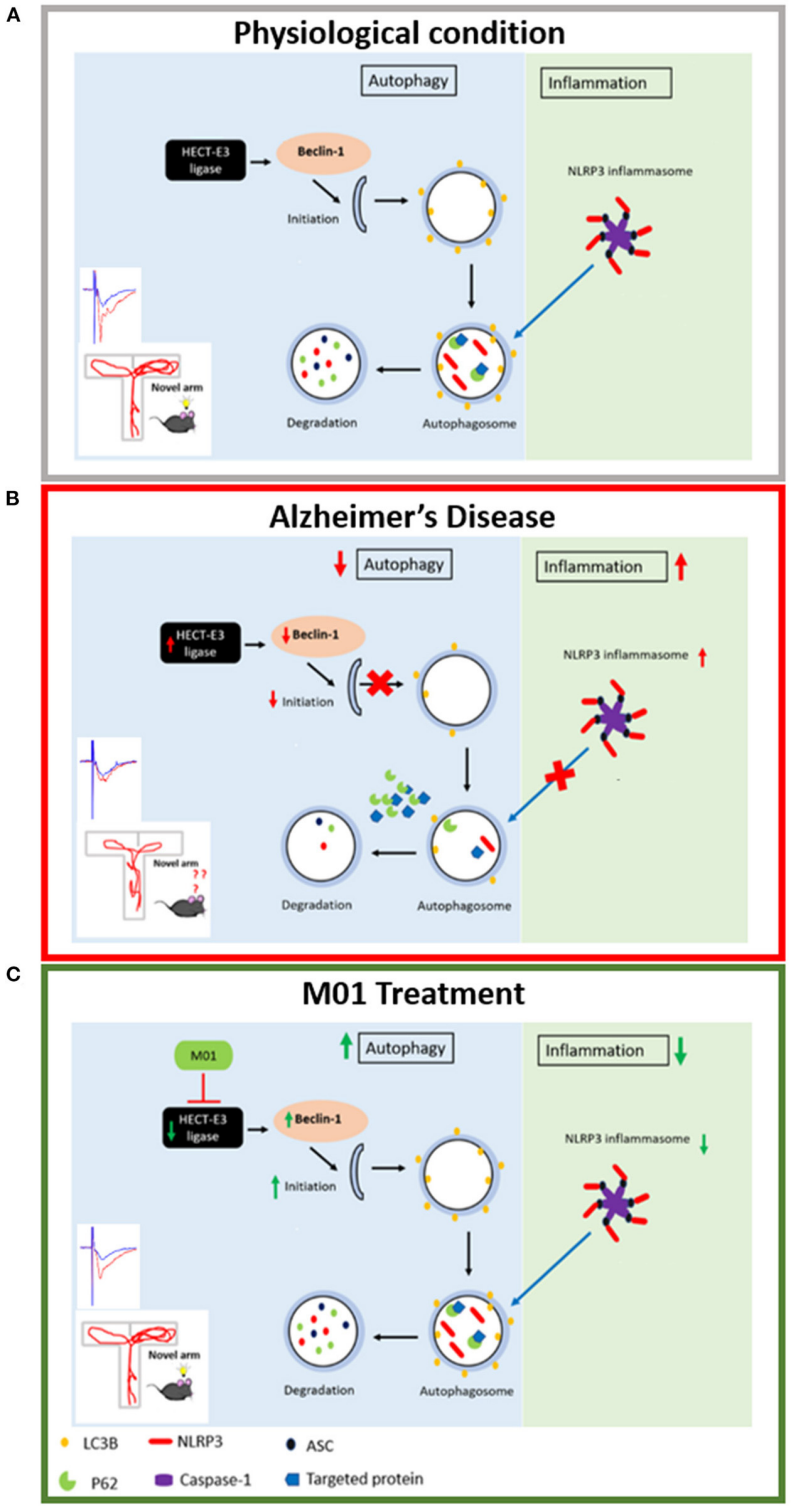
Schematic representation of the relationship between autophagy process and NLRP3 inflammasome. **(A)** Physiological condition: Under physiological condition, autophagy and NLRP3 inflammasome pathways are in a homeostatic status. **(B)** Alzheimer's disease condition: HECT-E3 ligase downregulates autophagy molecules, in turn, causes dysfunctional autophagy process and increase expression of the NLRP3 inflammasome in AD. **(C)** M01 treatment: M01 treatment rescued autophagy function and led to the degradation of the NLRP3 inflammasome proteins via autophagosomes.

The relationship between ubiquitination, autophagy, and the inflammatory response is dynamic and must be maintained at a homeostatic status for healthy cognitive functions in the brain (Figure 8A). Loss of this homeostasis leads to impaired memory performance in AD (Figure 8B), which can be rescued by administration of M01, the E3 ligase inhibitor (Figure 8C). Further experiments are necessary to better understand the function of individual HECT E3 ligase in enhancing autophagy processes and reducing NLRP3 inflammasome protein levels for the purpose of selecting the most promising candidates for the novel AD drug development.

## Data availability statement

The original contributions presented in the study are included in the article/Supplementary material, further inquiries can be directed to the corresponding authors.

## Ethics statement

The animal study was reviewed and approved by Taiwan Ministry of Science and Technology Guidelines for Animals' Ethical Treatment and Institutional Animal Care and Use Committee of Tzu Chi University, Taiwan.

## Author contributions

IL and PS designed the experiments and wrote the manuscript. SJ assisted in behavioral experiments, performed immunofluorescence staining, and helped write manuscript. YY provided E3 ligase inhibitor (M01), advised in experimental design, and manuscript revision. H-JH performed and analyzed protein docking experiments. TP and PV assisted in treatment and animal experiments. C-CC advised on experimental design and hypothesis. All authors read and approved the final manuscript.

## Funding

This work was funded by the Ministry of Science and Technology (MOST), Taiwan (MOST-107-2410-H320-001-MY3, MOST-110-2410-H-320-004-MY2), and Buddhist Tzu Chi Medical Foundation (TCMF-SP-108-04). This work also received partial financial support from the TMU Research Center of Cancer Translational Medicine from the Featured Areas Research Center Program within the framework of the Higher Education Sprout Project by the Ministry of Education (MOE) in Taiwan, and the Ministry of Health and Welfare (Health and Welfare Surcharge of Tobacco Products grant MOHW111-TDU-B-221-014013).

## Conflict of interest

The authors declare that the research was conducted in the absence of any commercial or financial relationships that could be construed as a potential conflict of interest.

## Publisher's note

All claims expressed in this article are solely those of the authors and do not necessarily represent those of their affiliated organizations, or those of the publisher, the editors and the reviewers. Any product that may be evaluated in this article, or claim that may be made by its manufacturer, is not guaranteed or endorsed by the publisher.
