# Supplementary material for: Attenuation of HECT-E3 ligase expression rescued memory deficits in 3xTg-AD mice
Source: Front Aging Neurosci. 2022 Jul 29;14:916904. doi: 10.3389/fnagi.2022.916904 (PMC9372289; doi:10.3389/fnagi.2022.916904)
Supplement: Supplementary file 2 [file Image_1.pdf]

Supplementary Material

Supplementary Materials

Figure-Sup.1

A

Protein	PDB code	Docking Score
WWP1-HECT	1ND7	-8.12
NEDD4-HECT	5C91	-8.11
HUWE1-HECT	3H1D	-8.01
ITCH-HECT	3TUG	-7.27
NEDD4L	5HPK	-6.91

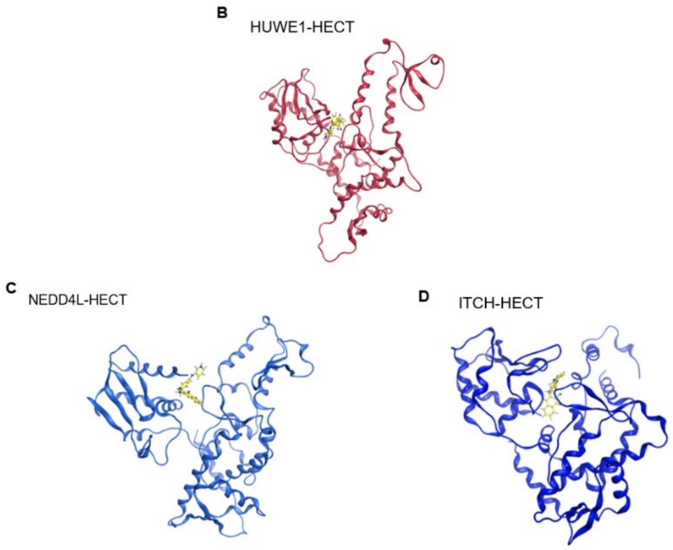

Fig.sup.1: Protein docking between different HECT-E3 ligases and M01. (A) Affinity score between HECT-E3 ligases and M01. PDB structure of E3 ligases and M01 (yellow) docking **(B)** HUWEI-HECT (red), **(C)** NEDD4L-HECT (light blue), **(D)** ITCH-HECT (dark blue)
